# Inflammatory Markers and Dyslipidemia in Patients with Oral Lichen Planus: A Case–Control Study

**DOI:** 10.3390/diagnostics15212783

**Published:** 2025-11-03

**Authors:** Pablo García-Roza, María García-Pola

**Affiliations:** Department of Surgery and Medical-Surgical Specialties, Faculty of Medicine and Sciences of the Health, Oviedo University, 33006 Oviedo, Spain

**Keywords:** oral lichen planus, biomarker, neutrophil, lipid-modifying agent, dyslipidemia, cholesterol, triglycerides, high-density-lipoprotein cholesterol, low-density-lipoprotein cholesterol, oral lichenoid reaction

## Abstract

**Background**: Lichen planus is a chronic inflammatory disease of multifactorial origin. **Objective**: The aim of study was to evaluate biomarker parameters of inflammation and dyslipidemia in oral lichen planus (OLP). **Methods**: Patients diagnosed clinically and histopathologically with OLP and health controls were matched by age and gender. In both groups, lipid and inflammatory parameter profiles were collected through blood tests. Univariate and multivariate regression analysis was performed to evaluate the association between dyslipidemia criteria and lipid-modifying agents and OLP adjusted for sex, age, tobacco use, and alcohol consumption. **Results**: The study included 252 subjects. The prevalence of dyslipidemia was 92 (73.02%) in OLP patients and 75 (59.52%) in the control group, with statistically significant differences [Odds Ratio (OR): 1.84 Confidence interval (CI) (1.08–3.12); *p* = 0.024)], while for lipid-modifying agent users it was 45 (35.71%) and 18 (14.29%), respectively [OR: 3.33 (1.79–6.18); *p* = 0.000)]. Drinkers with OLP had higher inflammatory response in the systemic immune–inflammation index (*p* = 0.013), systemic inflammation response index (*p* = 0.015), and pan-immune inflammation value (PIV) (*p* = 0.011). PIV was found to be higher in oral forms than in extraoral forms (*p* = 0.036). Multivariate analysis showed that patients being treated with lipid-modifying agents were three times more likely to be suffering from OLP (OR: 3.05;CI: 1.57–6.12, *p* = 0.001). **Conclusions**: The multivariate study showed that OLP is associated with lipid-modifying agents and not with dyslipidemia. The study included lipid inflammation markers that provide data on the behavior of OLP; however, the suggested inflammatory biomarkers do not offer a diagnostic alternative.

## 1. Introduction

Lichen planus (LP) is a chronic inflammatory disease of the skin, mucous membranes, and skin appendages. Its oral location (OLP) is considered a potentially malignant disorder [1]. The overall prevalence of OLP is 1.01% [2] and malignant transformation occurs in 1.43% of cases [3].

The origin of OLP remains unclear but is thought to be multifactorial. Several studies indicate that extrinsic or intrinsic antigens stimulate dendritic cells, especially Langerhans cells, which are responsible for presenting the antigens and interacting with T lymphocytes [4]. Various cell attraction mechanisms—including mast cells and macrophages derived from blood monocytes—and the production of cytokines, chemokines, and enzymes trigger apoptosis and pyroptosis of the basal cells in the mucosal epithelium, causing their membranes to rupture [4,5].

The most common clinical forms of OLP are papular–reticular and atrophic–erosive. Studies suggest that there are no differences in the presence of the sub-basal inflammatory component that defines OLP [6]. However, erosive forms have been reported to present a greater acute inflammatory component [7] and greater expression of oxidative stress [8].

OLP is associated with various diseases, such as autoimmune, endocrine, infectious, and cardiovascular diseases (CVD) [9]. There have been few case–control studies of CVD in OLP, and no differences have been found [10,11]. The difficulty in covering all types of CVD [12] means that the CVD literature more commonly deals with risk factors in OLP, such as hypertension, diabetes, and dyslipidemia [9,13]. Based on clinical histories, some authors have reported higher prevalence in OLP patients (compared to a control group) for hyperlipidemia [14], while others have not, with similar situations for hypercholesterol [15] and dyslipidemia [16,17]. 

Dyslipidemia is defined by serum levels meeting one of the following four criteria: total cholesterol (TC) level 200 mg/dL or higher; triglyceride (TG) of 150 mg/dL or higher; low-density lipoprotein cholesterol (LDL-C) of 130 mg/dL or higher, or high-density lipoprotein cholesterol (HDL-C) less than 40 mg/dL [18]. Because just one of these components can indicate dyslipidemia, and because of possible ethnic and dietary habits, the global prevalence of dyslipidemia varies widely. For example, for high LDL-C, the prevalence is between 30.7% and 63.1% of the adult population [19,20].

Patients with LP may have a higher probability of presenting dyslipidemia [21,22,23], with higher levels of TC, TG, LDL-C, and lower levels of HDL-C [24,25,26]. However, not all studies have corroborated these findings in a statistically significant manner, either in patients with cutaneous LP [27,28,29] or OLP [17,30]. Patients with LP have been reported to have higher levels of TC, TG, and LDL-C but similar levels of HDL-C to a control group [31,32]. Lopez Jornet et al. [33] found differences between OLP patients and a control group for Castelli’s atherogenic index (TC/HDLC) and for HDL-C but did not report differences in mean values of TG, TC or LDL-C. In contrast, Aniyan et al. [21] found differences only in values of TG, and Mehdipour et al. [34] found statistical differences in TG and TC, which were higher in patients with OLP than in a control group. Furthermore, Ozbagcivan et al. [11] found greater impairment of lipid metabolism among patients with oral LP involvement than those with cutaneous or associated involvement. 

Blood cells are essential for the pathogenetic development of inflammation and the immune response. Currently used biomarkers indicating inflammation and the prognosis of some diseases include indices based on leukocytes (neutrophil-to-lymphocyte ratio, NLR) and platelets (platelet-to-lymphocyte ratio, PLR) are easily calculated through a routine blood test [35]. Other indices that have emerged as potential biomarkers include three or four blood cell types, such as the systemic immune inflammation index (SII), system inflammation response index (SIRI), and pan-immune–inflammation value (PIV). These inflammatory parameters have been evaluated in cancer and precancerous oral conditions [36]. They have also been used to distinguish between different autoimmune bullous disorders [35], and between psoriasis and LP, with the former showing higher values in NLR, PLR, and SII indices [37]. Other studies have highlighted statistically significant differences in NLR values for LP [38,39], while conversely, no statistically significant differences were observed in another sample [40], or between an OLP and a control group [33]. Another parameter that indicates inflammation and oxidative stress in the prognosis of cardiovascular disorders is the ratio of monocyte counts to HDL-C (MHR) [41,42]. Smokers have been reported to have a significantly higher MHR than non-smokers [43]. This routine lipidic/inflammatory marker has been shown to be higher in dermatological patients with vitiligo [44], psoriasis [45], and LP than in a control group [46], but it has not been specifically analyzed in OLP patients. 

However, there are factors that could bias the results, such as data collection from databases rather than directly from the patient. For example, retrospective studies of recurrent aphthous stomatitis indicate greater differences in NLR than prospective studies [47].

Due to the possible link between dyslipidemias and OLP, with different results in the studies noted above, heterogeneity in data collection and interpretation, and the need to determine inflammatory involvement, the objectives of this prospective study were as follows: (1) to evaluate the biomarker parameters of inflammation and dyslipidemia in patients with OLP and compare them with a control group, and (2) to determine the association between dyslipidemia and its treatment in OLP patients. 

## 2. Material and Methods

### 2.1. Study Design

This study was designed according to the Strengthening Reporting of Observational Studies in Epidemiology (STROBE) guidelines for case–control studies (Table S1) [48]. 

### 2.2. Setting

The study was conducted between April 2023 and July 2025, in compliance with the data protection requirements of the Declaration of Helsinki and following approval by the Ethics Committee of Asturias (No. 2023/140). Patients were consecutively included in the OLP comorbidity study protocol undertaken at the Department of Oral Medicine in the University of Oviedo (Spain). 

### 2.3. Participants

#### 2.3.1. Inclusion Criteria

- Oral lichen planus patients. Patients over 18 years old, diagnosed with OLP, who had not received treatment for OLP in the previous 6 months. The World Health Organization (WHO) diagnostic criteria and later modifications were considered [49,50,51]. The clinical criteria were as follows: more or less bilateral presence of papules and reticulum, atrophy–erosion, plaques, and bullae associated with the papular–reticulum. The histopathological criteria were assessed via biopsy and included the following: cellular lymphocytic infiltration localized to the superficial part of the connective tissue; liquefactive degeneration in the basal layer of the epithelium; and the absence of epithelial dysplasia.
- Control group. Age and sex-matched subjects were selected consecutively in the same period of time. The control group consisted of subjects with normal oral mucosa who attended regular checkups, and subjects with benign oral lesions such as traumatic ulcers, mucoceles, and denture hyperplasia.

#### 2.3.2. Exclusion Criteria for OLP and Control Group

Those under 18 years old; pregnant or breastfeeding; oral lichenoid reaction; oral epithelial dysplasia; OLP associated with oral cancer at the time of diagnosis; and patients treated with radiotherapy or cancer chemotherapy. Oral lichenoid and dysplasia reactions were excluded to prevent confusion between OLP and oral lichenoid reactions in its nomenclature [1].

### 2.4. Variables and Measurement 

Demographic data were collected, including sex, age, tobacco use, and alcohol consumption. Tobacco use was recorded as smokers, non-smokers, and ex-smokers (who had stopped smoking at least 6 months prior) [52]. Alcohol use was defined as the consumption of at least 1 unit (U) of alcohol per day (i.e., 1 U of wine, beer, or spirits) or the equivalent over a weekend [52]. 

Clinical histories were taken from all patients and control subjects at the initial visit, including any medications such as lipid-modifying agents (ATC C10) [53]. This was followed by an examination of their oral cavity. 

Dyslipidemia was assessed in accordance with the guidelines from the National Cholesterol Education Program Adult Treatment Panel III (NCEP-ATP III) [18] as meeting one of the following four criteria: total cholesterol (TC) level 200 mg/dL or higher; TG of 150 mg/dL or higher; LDL-C of 130 mg/dL or higher, or HDL-C less than 40 mg/dL.

The clinical forms of OLP were divided into papular–reticular and atrophic–erosive lesions. The number of intraoral locations was recorded either at 2 or at 3 or more locations, as well as whether there were any extraoral locations. Duration of OLP prior to diagnosis was recorded as less than 6 months or more than 6 months.

### 2.5. Bias

The risk of bias was reduced given the consecutive selection of patients until reaching the desired sample size.

### 2.6. Study Size

**Figure 1:**
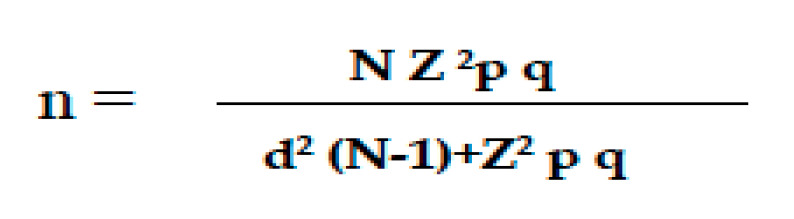
Formula for calculating the sample size. n: sample size; N: total population size; Z: statistical parameter corresponding to the desired confidence level of 95%; d: precision, in this case, 3%; p: probability of the event occurring (5%; 0.05); q: probability of the event not occurring (1-p; 0.95).

The sample size for OLP was calculated from a representative sample of the adult population over 18 years of age in the region of Asturias. A precision of 3% (d), a certainty of 95% (Z = 1.96), an expected proportion of 5% (0.05) and with q (1-p, 0.95) were considered (Figure 1) [54].

### 2.7. Quantitative Variable

After initial examination of the mouth, a blood test was requested and taken and analyzed on the day the biopsy was scheduled. The following parameters were considered in the study: red blood cells, hemoglobin, white blood cells, neutrophils, lymphocytes, monocytes, eosinophils, basophils, platelets, volume of platelets, TC, TG, LDL-C, and HDL-C. Values were recorded in 10^9^/L, g/dL/, fl, and mg/dL. In addition, the following ratios were calculated (Table 1): neutrophil/lymphocyte ratio (NLR); platelet/lymphocyte ratio (PLR); neutrophil/platelet ratio (NPR); lymphocyte/monocyte ratio (LMR); TC/HDL ratio; LDL/HDL ratio; monocytes/HDL ratio; TG/HDL ratio; systemic immune–inflammation index (SII); systemic inflammation response index (SIRI); and pan-immune inflammation value (PIV).

### 2.8. Statistical Analyses

Data were recorded in an Excel file. Statistical analyses were performed using R (R Development Core Team, Vienna, Austria), version 4.4.3. First, descriptive statistics were calculated for each variable, providing absolute and relative frequency distributions for qualitative variables, and measures of position and dispersion for quantitative variables, along with means and medians. Differences between qualitative variables were then assessed using Pearson’s chi-square test or Fisher’s exact test for qualitative variables, depending on whether the hypothesis regarding expected frequencies was expected. Student’s t-test or the Wilcoxon test were used to compare the differences in quantitative variables for independent samples depending on whether normality was confirmed. 

For the analytical study, variables were considered dichotomously (presence/absence, or yes/no) or, for smoking habits, as smokers, ex-smokers, or non-smokers. Age was classified into two categories, <60 years old and ≥60 years old. Differences between categorical variables were assessed using either Pearson’s chi-square test or Fisher’s exact test. Where there were 3 or more groups, ANOVA or the Kruskal–Wallis test was used, again depending on normality and homoscedasticity.

The relationships between variables were quantified by crude ratios. The multivariate model was created incorporating the variables: age (<60≥), sex (female/male), tobacco (no/yes/-ex), alcohol (no/yes), lipid-modifying agents (no/yes), and presence of dyslipidemia (no/yes). Results were considered statistically significant where *p* < 0.05.

## 3. Results

### 3.1. Descriptive and Univariate Analysis of the Sample

#### 3.1.1. Demographic Variables

The study included 126 OLP patients and 126 subjects without OLP as a control group. Of these subjects—matched by sex and age—188 were female (74.6%) and 64 were male (25.4%). The age range was 19 to 85 years, and the average age was 59.62 ± 12.42. The average age was 61.26 ± 11.86 years for women and 54.84 ± 13.09 years for men, with statistically significant differences (*p* = 0.011). 

There were no statistically significant differences between patients with OLP and the control group in tobacco or alcohol use, with non-smokers and non-drinkers predominating (Table 1).

#### 3.1.2. Clinical Form, Extension, Extraoral Location, and Duration

Two-fifths of the OLP patients (52; 41.3%) exhibited the papular–reticular clinical form and 74 (58.7%) the atrophic–erosive. Two-thirds (83; 65.9%) presented three or more locations of intraoral involvement, while 15 (11.9%) showed extraoral involvement (Table 1). 

OLP lasting more than 6 months was recorded in 82 patients (65.08%). The proportion of such cases was greater in men (*p* = 0.026), in atrophic–erosive forms (*p* = 0.009), and in cases with greater extension (*p* = 0.002).

#### 3.1.3. Biochemical and Chronic Inflammatory Parameters by Group

The laboratory findings from the participants by group are shown in Table 2. Values for red blood cells, hemoglobin, white blood cells, platelets, and platelet volume were similar between the OLP and control groups, with no statistically significant differences. There was a similar pattern with the ratios of inflammation.

Only the mean TC levels were pathological in both OLP patients and the control group (203.18 mg/dL vs. 201.10 mg/dL), with no statistically significant differences (*p* = 0.658). TG were higher and LDL-C were lower in OLP patients than in the control group, although the differences were not statistically significant. HDL-C was statistically significantly higher in OLP patients than in control group subjects (*p* = 0.016), but in neither group were the levels pathological. The LDL/HDL ratio was lower in OLP patients than in the control group (*p* = 0.028).

#### 3.1.4. Biochemical and Chronic Inflammatory Parameters in OLP Patients

There were statistically significant differences by sex, the following indicators being higher in men with OLP than women: red blood cells (*p* < 0.001), hemoglobin (*p* < 0.001), white blood cells (*p* = 0.008), neutrophils (*p* = 0.002), monocytes (*p* = 0.002), eosinophils (*p* = 0.027), and platelet volume (*p* = 0.022), (Table 3). The following were also higher in men with OLP than women: NLR, SIRI, PIV, and the TG/HDL ratio. TC and HDL-C counts were higher in women with OLP.

There were no statistically significant differences between current smokers, ex-smokers, and non-smokers in patients with OLP in any of the variables.

There were statistically significant differences according to alcohol consumption. Patients who drank had higher white blood cell (*p* = 0.011), neutrophil (*p* = 0.009), monocyte (*p* = 0.035), eosinophil (*p* = 0.031), and platelet counts (*p* = 0.049). They also had statistically significantly higher scores in SII (*p* = 0.013), SIRI (*p* = 0.015), and PIV (*p* = 0.011) (Table 4).

Patients with papular–reticular forms had statistically significantly higher platelet counts (*p* < 0.001) (Table S3).

Those with oral-only forms had higher monocyte counts (*p* = 0.006). They also had a higher neutrophil/platelet ratio (*p* = 0.008), higher SIRI (*p* = 0.001), and higher PIV (*p* = 0.036) (Table S4).

#### 3.1.5. Dyslipidemia and Treatment with Lipid Modified Agents

Almost three-quarters (92; 73.02%) of the OLP patients exhibited dyslipidemia, compared to under two-thirds (75; 59.52%) in the control group, a statistically significant difference [OR: 1.840, 95%CI (1.083–3.127); *p* = 0.024). OLP patients received statistically significantly more treatments with lipid-modifying agents than the control group [OR: 3.33, 95%CI (1.79–6.18), (*p* < 0.001)] (Table 1).

Differences in the presence of a single dyslipidemia parameter between the OLP and the control group were not statistically significant (Table 1) for TC *p* = 0.611, TG *p* = 0.481, LDL-C *p* = 1, and HDL-C *p* = 0.79.

### 3.2. Multivariate Analysis

A multivariate binary logistic regression model was specified, incorporating the variables age, sex, tobacco, alcohol, lipid-modifying agents, and presence of dyslipidemia using a stepwise selection algorithm. The results of the model indicated that OLP patients were more likely to use lipid-modifying agents [OR = 3.05, 95%CI (1.57–6.12); *p* = 0.001] (Table 5).

## 4. Discussion

OLP is an inflammatory mucocutaneous disease that has been suggested to be comorbid with other diseases [9]. The present study, based on the results of patient blood testing, shows that suffering from dyslipidemia was associated with the presence of OLP (*p* = 0.024). Although initially this association was observed in patients with OLP, it should be noted that the dyslipidemia was due to the presence of two or more risk factors rather than a single elevated factor of TC, TG, LDL-C or HDL-C; in the multivariate study, dyslipidemia was not found to be a risk factor for OLP. Conversely, patients being treated with lipid-modifying agents were three times more likely to be suffering from OLP.

When interpreting data from other studies, regardless of whether they compare values between cases and controls or identify statistically significant differences between them, it is essential to determine whether the values are sufficiently pathological to define hyperlipidemia or dyslipidemia. 

The present study found pathological TC values in both the OLP group and the control group, as some authors have also reported [11,30,33]. Other studies, however, despite reporting differences in values between case and control groups, have not found levels meeting the criteria for dyslipidemia [29,32].

Values for TG were normal in both sample groups, which is consistent with some studies [27,30,33,34], but not with others that have reported higher values in OLP patients compared to the normal range in control groups [11,21,29,32]. 

There is also no uniform pattern of higher pathological levels of LDL-C in patients with OLP [11,27]. Some studies, including ours, have even reported lower levels of LDL-C in OLP patients than control groups [21,27,33,34]. 

Nahidi et al. [23] showed that patients with oral involvement had higher HDL-C levels than patients with other LP locations, and we have confirmed this. Previous studies have highlighted that all subjects, both cases and controls, exhibit HDL-C levels above 40 mg/dL [11,21,27,29,30,32,33,34], although HDL-C values are commonly lower in OLP patients than in control groups [11,21,30,33,34]. This is our study’s most important finding, since the OLP patients exhibited significantly higher levels of HDL-C and higher LDL-C/HDL-C ratios than the control group. This observation is difficult to explain, although it was also reported by the aforementioned authors. It could be down to the greater number of patients treated and the efficacy of treatment for dyslipidemia in patients with OLP, who are advised to go on a diet for cholesterolemia, hypertriglyceridemia, or mixed hyperlipidemia [55]. This was reported by Polić et al. [28] in a study where, after OLP patients were put on a diet, there was a statistically significant reduction in TC, TG and LDL-C levels, and an increase in HDL-C levels.

Recently, great importance has been placed on inflammatory biomarkers for diagnosis of some mucocutaneous diseases [35,44,45]. In the context of the overall analysis in the present study, the values of inflammatory markers did not indicate statistically significant differences between the OLP and control groups. These findings corroborate a prospective study with a larger sample size than López-Jornet et al. [33]. Despite this, the present study does indicate some novel aspects. Male OLP patients exhibited a greater inflammatory response in NLR, SIRI, and PIV, and lipid TG/HDL-C ratios than women. 

Looking at alcohol consumption, OLP patients who consumed alcohol had higher cell counts and significantly greater inflammatory responses in SII, SIRI, and PIV ratios. These data are consistent with the consideration of alcohol as a risk factor for cardiovascular disease [56,57], which is reflected in our study’s higher TG and TG/HDL-C ratio values.

Another contribution of the study is the significantly higher platelet presence in papular–reticular lichen forms than in atrophic–erosive lichen forms (*p* < 0.001), which could be explained by the proposed role of platelets in wound healing [58]. Furthermore, PIV was found to be higher in oral forms than in extraoral forms (*p* = 0.036). Platelets are agents that stimulate proinflammation through their membrane receptors and by recruiting monocytes, lymphocytes, adhesion molecules, and cytokines such as CCL5, cells, and molecules that are also involved in the pathogenesis of OLP [59]. Platelet membranes are enriched with specific surface receptors that precisely regulate their activation and modulate granule release, adapting to inflammatory processes. Therefore, functional dysregulation of platelets could contribute to the development of chronic inflammation [58]. However, our study, like the study by Lopez Jornet et al. [33], could not corroborate the greater platelet volume found in OLP samples reported by Yao et al. [60] and Zakaria et al. [61].

Greater neutrophil activation through calprotectin has been demonstrated when LP is located in the oral region compared to the cutaneous region. The findings from Khattab et al. [62] are consistent with our observations, in which patients with exclusively oral sites had higher values of NLR, NPR, SII, SIRI, and PIV than patients with involvement in other sites. Our findings also include a statistically significant difference in the monocyte/HDL ratio between patients with oral manifestations and patients with other manifestations. Monocytes are the main source of proinflammation, and HDL-C inhibits LDL-C oxidation, producing anti-inflammatory effects [44].

In summary, although factors such as being male and alcohol consumption are not risk factors for OLP, they do mark differences in the degree of inflammation and the tendency toward greater inflammation in the oral setting. However, in the multivariate analysis, only treatment with lipid-modifying agents was considered an associated factor. It should be noted that dyslipidemia was associated with more than one potential factor in the blood test, such as TC, TG, LDL-C, and HDL-C. 

One of the limitations of this study is that, as a single study, the results cannot be generalized. The relationship between patients with OLP and treatment for hyperlipidemia could be considered a lichenoid reaction; however, the patients in the study had bilateral involvement. Other limitations to consider are the heterogeneity of the control group and that other lifestyle variables or methods diagnostics related to dyslipidemia were not included.

## 5. Conclusions

In conclusion, the multivariate study showed that OLP is associated with lipid-modifying agents and not with dyslipidemia blood parameters. The study included lipid inflammation markers that provide data on the behavior of OLP; however, the classical diagnostic method remains the method of choice, and the suggested inflammatory biomarkers do not offer a diagnostic alternative.

## Figures and Tables

**Table 1:** Characteristics of patients from oral lichen planus (OLP) and control group. SD: standard deviation. LDL-C: low-density-lypoprotein cholesterol. HDL-C: high-density-lypoprotein cholesterol. Percentages in parentheses. Statistically significant association, *p* values with asterisk.

Variable		OLP	Control	*p* Value
Sex	Female	94 (74.6)	94 (74.6)	1
	Male	32 (25.4)	32 (25.4)	
Age	Mean (SD)	59.63 (12.4)	59.63 (12.4)	1
	<60 years	55 (43.65)	55 (43.65)	
	≥60 years	71 (56.35)	71 (56.35)	
Tobacco user	No	90 (71.43)	92 (73.02)	0.941
	Yes	21 (16.67)	19 (15.07)	
	Ex-smoker	15 (11.9)	15 (11.8)	
Alcohol drinker	No	93 (73.81)	105 (83.33)	0.065
	Yes	33 (26.19)	21 (16.67)	
Clinical form	Papular–reticular	52 (41.3)		
	Atrophic–erosive	74 (58.7)		
Oral location	2	43 (34.1)		
	≥3	83 (65.9)		
Extraoral location	No	111 (88.1)		
	Yes	15 (11.9)		
Duration prior to diagnosis	≤6months	44 (34.92)		
	>6months	82 (65.08)		
Lipid-modifying agents	No	81 (64.29)	108 (85.71)	<0.001 *
	Yes	45 (35.71)	18 (14.29)	
Dyslipidemia	No	34 (26.98)	51 (40.48)	0.024 *
	Yes	92 (73.02)	75 (59.52)	
Total Cholesterol ≥ 200 mg/dL	No	69 (54.76)	73 (57.94)	0.611
	Yes	57 (45.24)	53 (42.06)	
Triglycerides ≥ 150 mg/dL	No	105 (83.33)	109 (86.5)	0.481
	Yes	21 (16.67)	17 (13.5)	
LDL-Cholesterol ≥ 130 mg/dL	No	84 (66.67)	84 (66.67)	1
	Yes	42 (33.33)	42 (33.33)	
HDL-Cholesterol < 40 mg/dL	No	118 (93.65)	119 (94.44)	0.79
	Yes	8 (6.35)	7 (5.56)	

**Table 2:** Hematological parameters and ratios in oral lichen planus (OLP) patients and control group. Mean and standard deviation are in parentheses; median in brackets. LDL-C: low-density lypoprotein cholesterol. HDL-C: high-density lypoprotein cholesterol. SII: systemic immune inflammation index. SIRI: system inflammation response index. PIV: pan-immune–inflammation value. With asterisk: statistically significant.

Variable	OLP (Mean), [Median]	Control	*p*
Red blood cells (10^9^/L)	4.66 (±0.46) [4.58]	4.60 (±0.38) [4.59]	0.255
Hemoglobin (g/dL)	13.98 (±1.18) [14]	13.79 (±1.21) [13.7]	0.201
White blood cells (10^9^/L)	6.29 (±1.44) [6.2]	6.24 (±1.87) [6.10]	0.802
Neutrophils (10^9^/L)	3.36 (±1.08) [3.24]	3.27 (±1.52) [3.04]	0.577
Lymphocytes (10^9^/L)	2.19 (±0.65) [2.09]	2.22 (±0.7) [2.11]	0.714
Monocytes (10^9^/L)	0.49 (±0.17) [0.47]	0.49 (±0.18) [0.5]	0.894
Eosinophils (10^9^/L)	0.19 (±0.12) [0.17]	0.19 (±0.13) [0.17]	0.957
Basophils (10^9^/L)	0.05 (±0.04) [0.04]	0.07 (±0.02) [0.03]	0.243
Platelets (10^9^/L)	233.04 (±53.65) [223.00]	238.9 (±56.88) [235]	0.401
Volume (fl)	9.95 (±1.56) [10.00]	9.86 (±1.47) [9.95]	0.657
Neutrophil/Lymphocyte ratio	1.68 (±0.76) [1.48]	1.63 (±1.02) [1.38]	0.675
Platelet/Lymphocyte ratio	118.09 (±47.57) [104.57]	118.36 (±54.67) [105.33]	0.967
Neutrophils/Platelet ratio	0.02 (±0.02) [0.01]	0.02 (±0.01) [0.01]	0.201
Lymphocytes/Monocytes ratio	5.57 (±5.49) [4.34]	5.29 (±2.56) [4.96]	0.606
SII	393.98 (±206.86) [347.06]	393.70 (±307.48) [320.93]	0.993
SIRI	0.86 (±0.66) [0.73]	0.88 (±1.0) [0.60]	0.805
PIV	190.89 (±138.6) [159.81]	206.49 (±278.91) [127.06]	0.574
Total Cholesterol (mg/dL)	203.18 (±36.37) [203.00]	201.10 (±38.37) [196.00]	0.658
LDL-C (mg/dL)	118.13 (±32.47) [119.00]	121.96 (±36.0) [119.5]	0.377
Triglycerides (mg/dL)	101.93 (±43.42) [94.5]	100.20 (±49.30) [86.5]	0.768
HDL-C (mg/dL)	66.48 (±19.52) [65.00]	61.12 (±15.29) [58.00]	0.016 *
Total Cholesterol/HDL-C ratio	3.21 (±0.9) [3.10]	3.41 (±1.07) [3.32]	0.107
LDL-C/HDL-C ratio	1.92 (±0.76) [1.84]	2.15 (±0.9) [2.04]	0.028 *
Monocytes/HDL-C ratio	0.01 (±0.02) [0.01]	0.01 (±0.05) [0.01]	0.472
Triglycerides/HDL-C ratio	1.79 (±1.43) [1.42]	1.82 (±1.18) [1.48]	0.84

**Table 3:** Hematological parameters and ratios in oral lichen planus (OLP) by sex. Mean and standard deviation in parentheses; median in brackets. LDL-C: low-density lypoprotein cholesterol. HDL-C: high-density lypoprotein cholesterol. SII: systemic immune inflammation index. SIRI: system inflammation response index. PIV: pan-immune–inflammation value. Statistically significant association, *p* values with asterisk.

Variable	Female	Male	*p*
Red blood cells (10^9^/L)	4.54 (±0.4) [4.52]	4.98 (±0.45) [5.1]	<0.001 *
Hemoglobin (g/dL)	13.68 (±1.03) [13.8]	14.88 (±1.14) [15.1]	<0.001 *
White blood cells (10^9^/L)	6.06 (±1.27) [6.03]	6.97 (±1.71) [6.62]	0.008 *
Neutrophils (10^9^/L)	3.15 (±0.94) [3.04]	3.97 (±1.26) [3.71]	0.002 *
Lymphocytes (10^9^/L)	2.19 (±0.65) [2.11]	2.16 (±0.69) [1.96]	0.827
Monocytes (10^9^/L)	0.46 (±0.16) [0.46]	0.57 (±0.19) [0.56]	0.002 *
Eosinophils (10^9^/L)	0.18 (±0.12) [0.16]	0.23 (±0.13) [0.22]	0.027 *
Basophils (10^9^/L)	0.05 (±0.03) [0.04]	0.05 (±0.05) [0.04]	0.805
Platelets (10^9^/L)	238.39 (±50.84) [234.00]	217.31 (±59.23) [204]	0.055
Volume (fl)	9.76 (±1.52) [9.75]	10.49 (±1.59) [10.4]	0.022 *
Neutrophil/Lymphocyte ratio	1.58 (±0.74) [1.43]	1.97 (±0.78) [1.82]	0.011 *
Platelet/Lymphocyte ratio	119.73 (±48.18) [107.56]	113.26 (±46.15) [103.52]	0.509
Neutrophils/Platelet ratio	0.02 (±0.03) [0.01]	0.02 (±0.01) [0.02]	0.721
Lymphocytes/Monocytes ratio	5.42 (±2.64) [4.72]	6.01 (±10.01) [3.98]	0.745
SII	382.95 (±213.5) [337.29]	426.38 (±185.32) [399.44]	0.307
SIRI	0.74 (±0.54) [0.67]	1.20 (±0.84) [1.06]	0.006 *
PIV	175.42 (±136.63) [146.7]	236.35 (±136.36) [206.99]	0.031 *
Total Cholesterol (mg/dL)	207.39 (±35.96) [206.00]	190.81 (±35.24) [190.50]	0.025 *
LDL-C (mg/dL)	120.96 (±32.56) [120.00]	109.84 (±31.24) [110.00]	0.095
Triglycerides (mg/dL)	97.36 (±35.67) [92.00]	115.34 (±59.45) [103.00]	0.114
HDL-C (mg/dL)	69.34 (±17.63) [69.00]	58.06 (±22.49) [55.00]	0.004 *
Total Cholesterol/HDL-C ratio	3.11 (±0.78) [3.00]	3.5 (±1.14) [3.22]	0.08
LDL/HDL ratio	1.86 (±0.71) [1.80]	2.104 (±0.89) [1.94]	0.121
Monocytes/HDL-C ratio	0.01 (±0.01) [0.01]	0.02 (±0.03) [0.01]	0.129
Triglycerides/HDL-C ratio	1.55 (±0.88) [1.35]	2.47 (±2.31) [1.56]	0.035 *

**Table 4:** Hematological parameters and ratios in oral lichen planus (OLP) by alcohol consumer. Mean and standard deviation in parentheses; median in brackets. LDL-C: low-density lypoprotein cholesterol. HDL-C: high-density lypoprotein cholesterol. SII: systemic immune inflammation index. SIRI: system inflammation response index. PIV: pan-immune–inflammation value. Statistically significant association, *p* values with asterisk.

Variable	No (*n* = 93)	Yes (*n* = 33)	*p*
Red blood cells (10^9^/L)	4.64 (±0.42) [4.57]	4.71 (±0.54) [4.62]	0.464
Hemoglobin (g/dL)	13.98 (±1.18) [13.9]	13.99 (±1.17) [14.1]	0.966
White blood cells (10^9^/L)	6.06 (±1.26) [6.00]	6.93 (±1.72) [6.75]	0.011 *
Neutrophils (10^9^/L)	3.18 (±0.91) [3.04]	3.87 (±1.35) [3.69]	0.009 *
Lymphocytes (10^9^/L)	2.17 (±0.64) [2.10]	2.23 (±0.7) [2.08]	0.672
Monocytes (10^9^/L)	0.47 (±0.17) [0.46]	0.55 (±0.19) [0.53]	0.035 *
Eosinophils (10^9^/L)	0.18 (±0.11) [0.15]	0.24 (±0.14) [0.26]	0.031 *
Basophils (10^9^/L)	0.04 (±0.04) [0.03]	0.05 (±0.04) [0.04]	0.523
Platelets (10^9^/L)	227.44 (±50.6) [220.00]	248.82 (±59.44) [245]	0.049 *
Volume (fl)	9.95 (±1.53) [10.00])	9.94 (±1.68) [10.0]	0.967
Neutrophil/Lymphocyte ratio	1.60 (±0.73) [1.41]	1.89 (±0.83) [1.82]	0.062
Platelet/Lymphocyte ratio	116.03 (±46.59) [103.68]	123.89 (±50.53) [105.46]	0.417
Neutrophils/Platelet ratio	0.02 (±0.03) [0.01]	0.02 (±0.01) [0.01]	0.208
Lymphocytes/Monocytes ratio	5.28 (±2.62) [4.50]	6.37 (±9.85) [4.00]	0.536
SII	366.89 (±192.94) [336.46]	470.33 (±228.0) [437.65]	0.013 *
SIRI	0.74 (±0.48) [0.62]	1.17 (±0.93) [0.9]	0.015 *
PIV	172.29 (±132.51) [144.22]	243.30 (±143.93) [200.11]	0.011 *
Total Cholesterol (mg/dL)	205.96 (±35.82) [204.00]	195.36 (±37.32) [199.00]	0.151
LDL-C (mg/dL)	121.3 (±32.88) [121.00]	109.21 (±30.0) [110.00]	0.066
Triglycerides (mg/dL)	98.47 (±36.34) [91.0]	111.67 (±58.6) [98.0]	0.232
HDL-C (mg/dL)	66.92 (±19.93) [64.00]	65.21 (±18.54) [67]	0.667
Total Cholesterol/HDL-C ratio	3.26 (±0.91) [3.21]	3.08 (±0.85) [2.97]	0.314
LDL-C/HDL-C ratio	1.98 (±0.81) [1.91]	1.76 (±0.59) [1.8]	0.116
Monocytes/HDL-C ratio	0.01 (±0.02) [0.01]	0.01 (±0.02) [0.01]	0.876
Triglycerides/HDL-C ratio	1.69 (±1.10) [1.42]	2.07 (±2.11) [1.41]	0.323

**Table 5:** Univariate and multivariate analysis of the association between dyslipidemia and treatment of hyperlipidemia in OLP patients and the control group. Adjusted for sex, age, tobacco, and alcohol consumer. Confidence interval (CI).

Variable	OLP *n* (%)	Control Group *n* (%)	OR Univariate (95% CI, *p* Value)	OR Multivariate (95% CI, *p* Value)
Sex			1 (0.56–1.76; *p* = 1)	1.18 (0.62–2.27, *p* = 0.609)
Female (%)	94 (50.0)	94 (50.0)		
Male (%)	32 (50.0)	32 (50.0)		
Age			1 (0.60–1.64; *p* = 1)	0.81 (0.47–1.41, *p* = 0.465)
<60 (%)	55 (50.0)	55 (50.0)		
≥60 (%)	71 (50.0)	71 (50.0)		
Tobacco			1.08 (0.62–1.87; *p* = 0.77)	1.17 (0.63–2.16, *p* = 628)
No	90 (49.5)	92 (50.5)		
Yes-ex	36 (51.5)	34 (48.6)		
Alcohol			1.77 (0.96–3.27; *p* = 0.065)	1.43 (0.72–2.88, *p* = 0.314)
No	93 (47.0)	105 (53.0)		
Yes-ex	33 (61.1)	21 (38.9)		
Lipid-modifying agents			3.33 (1.79–6.18; *p* = 0.000)	3.05 (1.57–6.12, *p* = 0.001)
No	81 (42.9)	108 (57.1)		
Yes	45 (71.4)	18 (28.6)		
Dyslipidemia			1.84 (1.08–3.12; *p* = 0.024)	1.32 (0.74–2.39, *p* = 0.350)
No	34 (40.0)	51 (60.0)		
Yes	92 (55.1)	75 (44.9)		

## Data Availability

The data manifested in this study are available upon request from corresponding author. To protect privacy, the data are not publicity available.

## Supplementary Materials

The following supporting information can be downloaded at: https://www.mdpi.com/article/10.3390/diagnostics15212783/s1, Table S1, STROBE Statement. Checklist of items that should be considered in reports of case–control studies. Table S2, Biomarkers and ratios evaluated in this study. Table S3, Hematological parameters and ratios in oral lichen planus by clinical form. Table S4, Hematological parameters and ratios in oral lichen planus by other location.

## Abbreviations

The following abbreviations are used in this manuscript:

NLR	Neutrophil-to-lymphocyte ratio
PLR	Platelet-to-lymphocyte ratio
NPR	Neutrophil to platelet ratio
LMR	Lymphocyte to monocyte ratio
SII	Systemic immune–inflammation index
SIRI	Systemic inflammation response index
PIV	Pan-immune inflammation value
LDL-CHDL-C	Low-density-lipoprotein to high-density-lipoprotein cholesterol
M/HDL-C	Monocytes to high-density-lipoprotein cholesterol
TC/HDL-C	Total cholesterol to high-density-lipoprotein cholesterol
TG/HDL-C	Triglycerides to high-density-lipoprotein cholesterol
