# Supplementary material for: Inflammatory Markers and Dyslipidemia in Patients with Oral Lichen Planus: A Case–Control Study
Source: Diagnostics (Basel). 2025 Nov 3;15(21):2783. doi: 10.3390/diagnostics15212783 (PMC12609926; doi:10.3390/diagnostics15212783)
Supplement: Supplementary file 1 [file diagnostics-15-02783-s001.zip › diagnostics-3896182-supplementary.pdf]

Table S1. Annex I. STROBE Statement—Checklist of items that should be included in reports of case-control studies

	Item No	Recommendation	Page No
**Title and abstract**	1	(*a*) Indicate the study's design with a commonly used term in the title or the abstract	1
		(*b*) Provide in the abstract an informative and balanced summary of what was done and what was found	1
**Introduction**			
Background/rationale	2	Explain the scientific background and rationale for the investigation being reported	1-3
Objectives	3	State specific objectives, including any prespecified hypotheses	3
**Methods**			
Study design	4	Present key elements of study design early in the paper	3
Setting	5	Describe the setting, locations, and relevant dates, including periods of recruitment, exposure, follow-up, and data collection	3
Participants	6	(*a*) Give the eligibility criteria, and the sources and methods of selection of participants	3
Variables	7	Clearly define all outcomes, exposures, predictors, potential confounders, and effect modifiers. Give diagnostic criteria, if applicable	4
Data sources/ measurement	8*	For each variable of interest, give sources of data and details of methods of assessment (measurement). Describe comparability of assessment methods if there is more than one group	3,4
Bias	9	Describe any efforts to address potential sources of bias	4
Study size	10	Explain how the study size was arrived at	4
Quantitative variables	11	Explain how quantitative variables were handled in the analyses. If applicable, describe which groupings were chosen and why	4
Statistical methods	12	(*a*) Describe all statistical methods, including those used to control for confounding	4
		(*b*) Describe any methods used to examine subgroups and interactions	
		(*c*) Explain how missing data were addressed	
		(*d*) If applicable, describe analytical methods taking account of sampling strategy	
		(*e*) Describe any sensitivity analyses	
**Results**			
Participants	13*	(a) Report numbers of individuals at each stage of study—eg numbers potentially eligible, examined for eligibility, confirmed eligible, included in the study, completing follow-up, and analysed	5,6. Table1,2
		(b) Give reasons for non-participation at each stage	
		(c) Consider use of a flow diagram	

Descriptive data	14*	(a) Give characteristics of study participants (eg demographic, clinical, social) and information on exposures and potential confounders	5,6<br>Table 2-7
		(b) Indicate number of participants with missing data for each variable of interest	
Outcome data	15*	Report numbers of outcome events or summary measures	4
Main results	16	(*a*) Give unadjusted estimates and, if applicable, confounder-adjusted estimates and their precision (eg, 95% confidence interval). Make clear which confounders were adjusted for and why they were included	5-12
		(*b*) Report category boundaries when continuous variables were categorized	
		(*c*) If relevant, consider translating estimates of relative risk into absolute risk for a meaningful time period	
Other analyses	17	Report other analyses done—eg analyses of subgroups and interactions, and sensitivity analyses	4-7<br>Table 8
**Discussion**			
Key results	18	Summarise key results with reference to study objectives	12
Limitations	19	Discuss limitations of the study, taking into account sources of potential bias or imprecision. Discuss both direction and magnitude of any potential bias	12,13
Interpretation	20	Give a cautious overall interpretation of results considering objectives, limitations, multiplicity of analyses, results from similar studies, and other relevant evidence	13,14
Generalisability	21	Discuss the generalisability (external validity) of the study results	14
**Other information**			
Funding	22	Give the source of funding and the role of the funders for the present study and, if applicable, for the original study on which the present article is based	No funding

Table S2. Biomarkers and ratios evaluated in this study.

Term of biomarker	Interpretation and formula
Neutrophil-to-lymphocyte ratio (NLR)	The ratio of absolute neutrophil count to absolute lymphocyte count
Platelet-to-lymphocyte ratio (PLR)	The ratio of absolute platelet count to absolute lymphocyte count
Neutrophil-to-platelet ratio (NPR)	The ratio of absolute neutrophil count to absolute platelet count
Lymphocyte-to-monocyte ratio (LMR)	The ratio of absolute lymphocyte count to absolute monocyte count
Systemic immune-inflammation index (SII)	The ratio of absolute neutrophil count x platelet to absolute lymphocyte count
Systemic inflammation response index (SIRI)	The ratio of absolute neutrophil count x monocyte to absolute lymphocyte count
Pan-immune inflammation value (PIV)	The ratio of absolute neutrophil count x platelet x monocyte to absolute lymphocyte count

Total cholesterol to high-density-lipoprotein cholesterol (TC/HDL-C)	The ratio of mean cholesterol level to mean high-density-lipoprotein cholesterol
Monocytes to high-density-lipoprotein cholesterol (M/HDL-C)	The ratio of absolute monocyte count to mean high-density-lipoprotein cholesterol
Triglycerides to high-density-lipoprotein cholesterol (TG/HDL-C)	The ratio of mean triglycerides level to mean high-density-lipoprotein cholesterol
Low-density-lipoprotein to high-density-lipoprotein cholesterol (LDL-C/HDL-C)	The ratio of mean low-density-lipoprotein to mean high-density-lipoprotein cholesterol

Table S3 Hematological parameters and ratios in oral lichen planus (OLP) by clinical form. Mean, and standard deviation, in parenthesis, median in brackets. LDL-C: Low-Density Lypoprotein Cholesterol. HDL-C High-Density Lypoprotein Cholesterol. SII: systemic immune inflammation index. SIRI: system inflammation response index. PIV: pan-immune-inflammation value. Statistically significant association, p values with asterisk.

Variable	Atrophic-erosive	Papular-reticular	p
Red blood cells (10^9/L)	4.61(±0.46) [4.54]	4.72(±0.45) [4.66]	0.168
Hemoglobin (g/dL)	13.96(±1.33) [13.85]	14.01(±0.93) [14.10]	0.811
White blood cells (10^9/L)	6.36(±1.6) [6.24]	6.19(±1.18) [6.14]	0.488
Neutrophils (10^9/L)	3.45(±1.23) [3.35]	3.23(±0.83) [3.05]	0.228
Lymphocytes (10^9/L)	2.15(±0.67) [2.01]	2.23(±0.64) [2.19]	0.495
Monocytes (10^9/L)	0.48(±0.16) [0.48]	0.50(±0.19) [0.47]	0.483
Eosinophils (10^9/L)	0.19(±0.12) [0.18]	0.19(±0.13) [0.16]	0.851
Basophils (10^9/L)	0.05(±0.04) (0.04]	0.05(±0.04) [0.04]	0.919
Platelets (10^9/L)	216.84(±48.54) [216.00]	256.1(±52.52) [255.50]	<0.001*
Volume (fl)	9.97(±1.66) [9.95]	9.92(±1.44) [10.05]	0.867
Neutrophil/Lymphocyte ratio	1.75(±.83) [1.62]	1.58(±0.65) [1.39]	0.208
Platelet/Lymphocyte ratio	113.38(±48.4) [100.71]	124.61(±46.05) [116.23]	0.198
Neutrophils/Platelet ratio	0.02(±0.02) [0.01]	0.02(±0.03) [0.01]	0.531
Lymphocytes/Monocytes ratio	5.86(±6.92) [4.34]	5.15(±2.23) [4.4]	0.41
SII	385.42(±214.41) [345.47]	406.16(±197.03) [355.70]	0.582
SIRI	0.85(±0.58) [0.75]	0.87(±0.76) [0.68]	0.852
PIV	190.68(±156.5) [163.93]	191.17(±109.65) [149.20]	0.984
Total Cholesterol (mg/dL)	199.81(±35.41) [197.5]	207.98(±37.51) [208.5]	0.216
LDL-C (mg/dL)	116.28(±30.98) [116.00]	120.77(±34.62) [120.5]	0.447
Triglycerides (mg/dL)	103.20(±39.67) [96.0]	100.12(±48.6) [89.0]	0.696
HDL-C (mg/dL)	65.74(±20.71) [63.5]	67.52(±17.84) [70]	0.617
Total Cholesterol/HDL-C ratio	3.17(±0.87) [3.05]	3.27(±0.94) [3.23]	0.571
LDL-C/HDL-C ratio	1.92(±0.77) [1.81]	1.92(±0.76) [1.92]	0.986
Monocytes/HDL-C ratio	0.01(±0.02) [0.01]	0.01(±0.01) [0.01]	0.251
Triglycerides/HDL-C ratio	1.80(±1.22) [1.43]	1.77(±1.7) [1.36]	0.92

Table S4 Hematological parameters and ratios in oral lichen planus (OLP) by other location. Mean, and standard deviation, in parenthesis, median in brackets. LDL-C: Low-Density Lypoprotein Cholesterol. HDL-C High-Density Lypoprotein Cholesterol. Statistically significant association, p values with asterisk. Statistically significant association, p values with asterisk.

Variable	NO (mean, median)	YES	P value
Red blood cells (10^9/L)	4.66(±0.46) [4.6]	4.60(±0.44) [4.54]	0.638
Hemoglobin (g/dL)	14.05(±1.16) [14]	13.52(±1.23) [13.8]	0.104
White blood cells (10^9/L)	6.38(±1.43) [6.30]	5.65(±1.4) [5.65]	0.067
Neutrophils (10^9/L)	3.42(±1.11) [3.29]	2.92(±0.74) [2.72]	0.095
Lymphocytes (10^9/L)	2.20(±0.65) [2.10]	2.05(±0.7) [2.06]	0.406
Monocytes (10^9/L)	0.51(±0.18) [0.49]	0.38(±0.12) [0.35]	0.006*
Eosinophils (10^9/L)	0.19(±0.12) [0.17]	0.20(±0.14) [0.17]	0.865
Basophils (10^9/L)	0.05(±0.04) [0.04)	0.04(±0.04) [0.03]	0.773
Platelets (10^9/L)	232.41(±197.0) [222.00]	237.67(±210.0) [242]	0.723
Volume (fl)	10.03(±1.55) [10.10]	9.36(±1.6) [9.00]	0.12
Neutrophil/Lymphocyte ratio	1.70(±0.78) [1.49]	1.56(±0.59) [1.44]	0.505
Platelet/Lymphocyte ratio	114.81(±42.34) [103.68]	141.69(±73.78) [110.22]	0.299
Neutrophils/Platelet ratio	0.02(±0.03) [0.01]	0.01(±0.0) [0.01]	0.008*
Lymphocytes/Monocytes ratio	5.54(±5.8) [4.31)	5.78(±2.19) [5.57]	0.767
SII	396.66(±212.27) [345.37]	374.11(±166.01) [391.56]	0.693
SIRI	0.89(±0.68) [0.74]	0.58(±0.26) [0.47]	0.001*
PIV	197.31(±143.82) [165.53]	143.35(±79.06) [116.30]	0.036*
Total Cholesterol (mg/dL)	202.70(±36.72) [203.00)	206.73(±34.63) [200.00]	0.689
LDL-C (mg/dL)	117.32(±32.69) [119.00)	124.13(±31.23) [126]	0.448
Triglycerides (mg/dL)	103.99(±44.35) [96.00)	86.67(±33.02) [83.00]	0.148
HDL-C (mg/dL)	66.63(±20.36) [64.00)	65.33(±11.97) [68.00]	0.725
Total Cholesterol/HDL-C ratio	3.21(±0.93) [3.09)	3.25(±0.61) [3.25]	0.858
LDL/HDL ratio	1.92(±0.79) [1.83)	1.95(±0.54) [1.85]	0.855
Monocytes/HDL-C ratio	0.01(±0.02) [0.02)	0.01(±0.0) [0.00]	0.027*
Triglycerides/HDL-C ratio	1.83(±1.5) [1.42)	1.82(±0.75) [1.48]	0.137
